# Supplementary material for: Dehydration entropy drives liquid-liquid phase separation by molecular crowding
Source: Commun Chem. 2020 Jun 26;3:83. doi: 10.1038/s42004-020-0328-8 (PMC9814391; doi:10.1038/s42004-020-0328-8)
Supplement: Supplementary file 2 — Supplementary Information [file 42004_2020_328_MOESM2_ESM.pdf]

[Supporting information]

Dehydration entropy drives liquid-liquid phase separation by molecular crowding

Sohee Park,[1] Ryan Barnes,[2] Yanxian Lin,[3] Byoung-jin Jeon,[4] Saeed Najafi,[2,5] Kris T. Delaney,[5] Glenn H. Fredrickson,[4,5,6] Joan-Emma Shea,[2,7] Dong Soo Hwang,[*, 1, 8] and Songi Han,[*, 2, 6]

[1] Division of Environmental Science and Engineering, Pohang University of Science and Technology (POSTECH), 77 Chengam-ro, Nam-gu, Pohang 37673, Republic of Korea

[2] Department of Chemistry and Biochemistry, University of California, Santa Barbara, CA 93106, United States

[3] Department of Biomolecular Science and Engineering, University of California, Santa Barbara, CA 93106, United States

[4] Materials Department, University of California, Santa Barbara, CA 93106, United States

[5] Materials Research Laboratory, University of California, Santa Barbara, California 93106, USA

[6] Department of Chemical Engineering, University of California, Santa Barbara, CA 93106, United States

[7] Department of Physics, University of California at Santa Barbara, Santa Barbara, California 93106, United States

[8] Division of Integrative Biosciences and Biotechnology, Pohang University of Science and Technology (POSTECH), 77 Chengam-ro, Nam-gu, Pohang 37673, Republic of Korea

[*] co-Corresponding authors

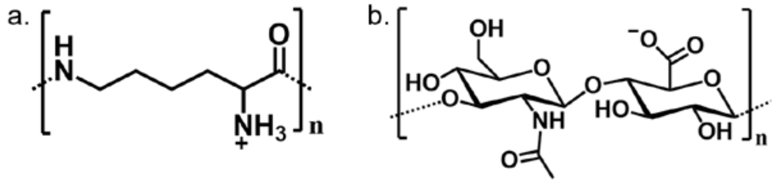

Supplementary Figure 1. (a) ε-poly-L-lysine (εPL) and (b) hyaluronic acid (HA)

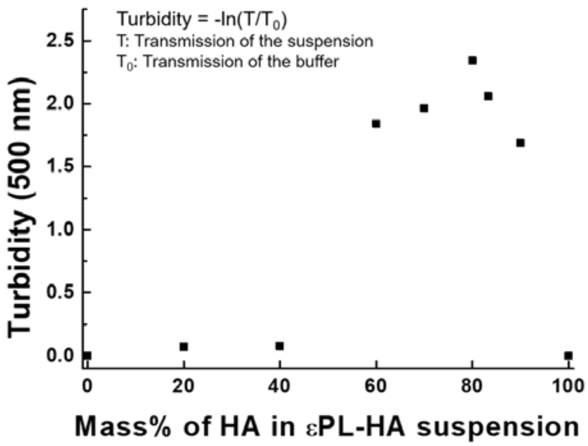

Supplementary Figure 2. Turbidity of microphase-separated coacervate suspensions at different εPL and HA ratio

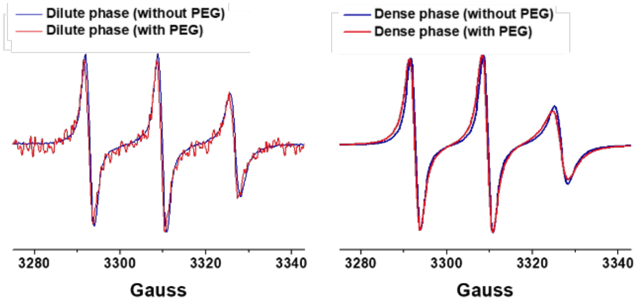

Supplementary Figure 3. EPR spectra of spin labeled εPL in both phases with (red) and without PEG (blue). Coacervate suspension with and without PEG were formed in 0.1 M, pH 5.0 sodium acetate, without additional NaCl.

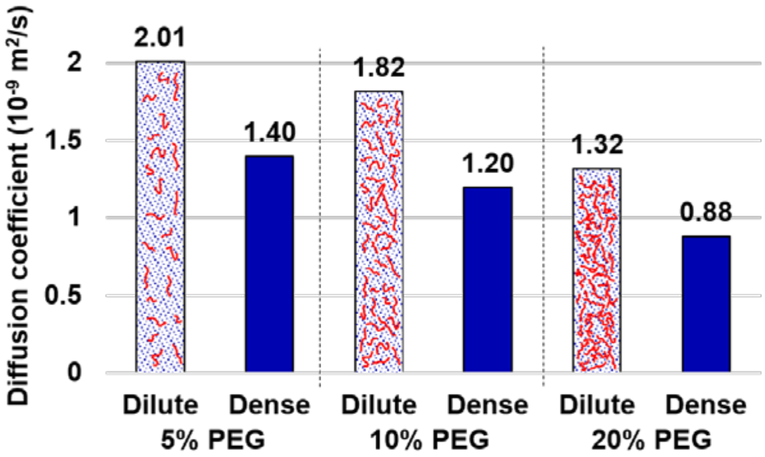

Supplementary Figure 4. Diffusion coefficients of interstitial water in the macro-separated dilute and dense phases under different PEG% concentration (5, 10, and 20%), without additional NaCl.

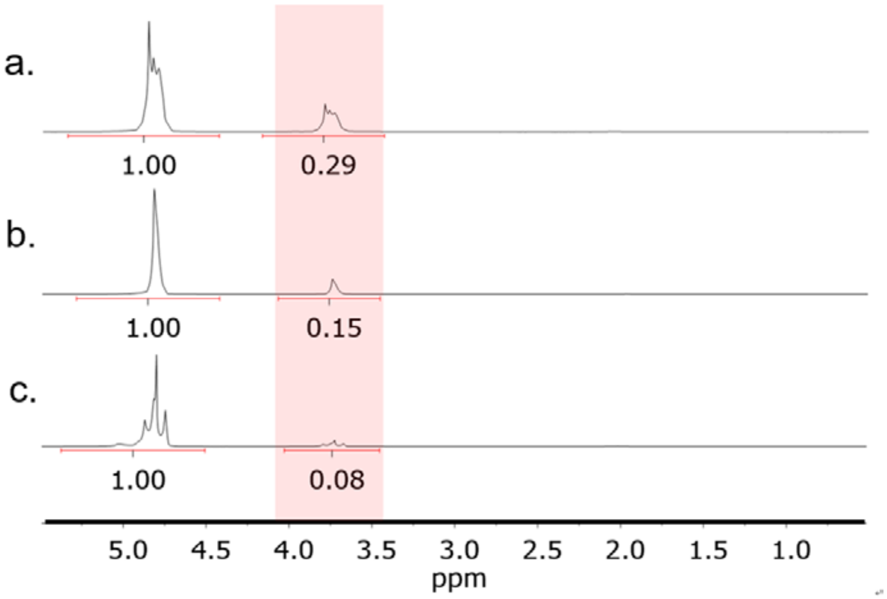

Supplementary Figure 5. ^{1}H PFG NMR spectra of the macro-separated dilute phase formed with different% of PEG. (A) 20%, (B) 10%, and (C) 5%

Supplementary Table 1. Calculated polyelectrolyte weight and concentration in dense coacervate phase.

		Water content (w/w%)	Polyelectrolytes content (w/w%)	Weight (g)	Volume (mL)
without PEG	dense	81.18±0.01	18.82±0.01	0.35±0.01	0.35±0.01
with PEG	dense	72.33±0.02	27.67±0.01	0.73±0.01	0.63±0.01

Water content in the macro-separated dense and dilute phase was measured using a moisture analyzer. We assumed polyelectrolyte content in the dense coacervate phase as (100% (w/w) - the water content). We measured the weight of the macro-separated dense phase by weighing the specific volume. The weight of the polyelectrolytes in dense phase was calculated as (the weight of the dense coacervate phase) × (the polyelectrolyte content (% (w/w)). The weight of the polyelectrolytes in the dilute phase was estimated as (300 mg of total polyelectrolytes we initially added – the weight of polyelectrolytes in the dense phase).

Supplementary Table 2. εPL-HA coacervate volume fraction was determined after the macro-separated phase separation under specific temperature. Coacervation in the presence of PEG happened in 0.1 M pH 5 sodium acetate, without additional NaCl.

	Coacervate volume fraction	
**Temperature (°C)**	Average	Standard deviation
RT	2.33	0.03
40	2.32	0.02
70	2.25	0.08

Supplementary Methods. Field-Theoretic Simulation (FTS)

1. Molecular model

In our model, the monomers interact through a soft repulsive excluded volume interaction $\beta U_{ex} = v_{ij}\delta(\vec{r})$ [1], where v_{ij} is the strength of the excluded volume interaction between the species. For the sake of simplicity, we assumed that polyelectrolytes and PEG monomers repel their own species with strength $v = v_{PP} = v_{CC}$. The cross excluded volume interaction between polyelectrolytes and PEG monomers is denoted by v_{PC}. Additionally, all the beads interact via a Coulomb potential screened by a uniform background dielectric of Bjerrum length l_B, $\beta U_{el} = \frac{l_B \sigma_i \sigma_j}{|\vec{r}|}$, with $l_B = \beta e^2/(4\pi\epsilon_0\epsilon_r)$, where ϵ_0 and ϵ_r are vacuum permittivity and relative dielectric strength of solvent, respectively, and $\beta = 1/k_BT$. Chain connectivity is enforced by a harmonic potential $\beta U_{bond} = \frac{3}{2b^2}\sum_{\alpha=1}^{n_P\&n_C}\sum_{j=1}^{N_P\&N_C}(|\vec{r}_{\alpha,j} - \vec{r}_{\alpha,j-1}|)^2$, where $\vec{r}_{\alpha,j}$ refers to the position of bead j on chain α, and b denotes the statistical segment length, n_P, n_C, N_P and N_C are the number of polyelectrolyte and PEG chains and their corresponding polymerization degree, repectively. The density and charge density of each bead smeared over a finite volume by convolving with a normalized Gaussian profile $\Gamma(\vec{r}) = (2\pi a^2)^{-3/2}exp(-|\vec{r}|^2/2a^2)$, where a indicates the smearing width [2]. The resulting potential interaction for this model is given by:

$$
\begin{aligned}
\beta\bar{U}(\vec{r}) =& \frac{3}{2b^2}\sum_{\alpha=1}^{n_P}\sum_{j=1}^{N_P}(|\vec{r}_{\alpha,j} - \vec{r}_{\alpha,j-1}|)^2 + \frac{3}{2b^2}\sum_{\alpha=1}^{n_C}\sum_{j=1}^{N_C}(|\vec{r}_{\alpha,j} - \vec{r}_{\alpha,j-1}|)^2 \\
&+ \frac{v}{2}\int d\vec{r}\,(\bar{\rho}_P(\vec{r}) + \bar{\rho}_C(\vec{r}))^2 + v_{PC}\int d\vec{r}\,\bar{\rho}_P(\vec{r})\bar{\rho}_C(\vec{r}) + \frac{l_B}{2}\int\int d\vec{r}\,d\vec{r}\,'\,\frac{\bar{\rho}_e(\vec{r})\bar{\rho}_e(\vec{r}\,')}{|\vec{r} - \vec{r}\,'|}
\end{aligned}
\tag{1}
$$

where $\bar{\rho}_P(\vec{r})$ and $\bar{\rho}_C(\vec{r})$ refers to the smeared microscopic density of polyelectrolytes and PEG, respectively, and $\bar{\rho}_e(\vec{r})$ is the microscopic charge density.

2. Field theory

The canonical partition function of the model specified in Eq.1, can be converted from an integral over particle configurations to a complex-valued statistical field theory, using standard techniques based on the Hubbard-Stratonovich transformation [3]:

$$
Z_c = Z_0 \int Dw_+ \int Dw_- \int D\varphi \;\exp\left(-H[w_+, w_-, \varphi]\right) \tag{2}
$$

where w_+ and w_- are auxiliary fields conjugate to the total density and the difference in the densities of the species, respectively, and ϕ is a fluctuating electrostatic potential and Z_0 is the partition function for an ideal gas of discrete Gaussian chains and self-interaction correction terms. The field-theoretic Hamiltonian is:

$$\begin{aligned} H[w_+, w_-, \varphi] =& \frac{1}{2v + v_{PC}} \int d\vec{r}\, [w_+(\vec{r})]^2 + \frac{1}{v_{PC}} \int d\vec{r}\, [w_-(\vec{r})]^2 + \frac{1}{2} \int d\vec{r}\, \frac{|\nabla\varphi(\vec{r})|^2}{4\pi l_B} \\ & - \sum_l n_l \ln Q_l[w_+, w_-, \varphi] \end{aligned} \tag{3}$$

where we have introduced a set of non-dimensionalized parameters for convenience. Q_l, is the partition function for a single chain type l, which can be polyelectrolytes or PEG, in a complex-valued external field. In general $Q[w_+, w_-, \varphi]$ can be computed using a Gaussian chain propagator:

$$Q[w_+, w_-, \varphi] = \frac{1}{V} \int d\vec{r}\, q_N(\vec{r}; [w_+, w_-, \varphi]) \tag{4}$$

The chain propagator for polyelectrolytes $q_N(\vec{r}; \psi)$ can be constructed from the following Chapman-Kolmogorov-type equation:

$$q_{j+1}(\vec{r}; \psi) = \left(\frac{3}{2\pi b^2}\right)^{3/2} \exp[-\psi_{j+1}(\vec{r})] \int d\vec{r}\,' q_j(\vec{r}\,'; \psi) \exp\left(-\frac{3|\vec{r} - \vec{r}\,'|^2}{2b^2}\right) \tag{5}$$

with $\psi_j = i\Gamma \star (\frac{w_+ + iw_-}{\sqrt{2}} + \sigma_j \varphi)$, where $i = \sqrt{-1}$ and σ_j refers to the charge of the monomer of the polyelectrolytes, and $\star$ denotes a spatial convolution. The initial condition is $q_0(\vec{r}; \psi) = exp[-\psi_0(\vec{r})]$. The crowder chains obey the similar equation with a field $\psi_c = i\Gamma \star (\frac{w_+ - iw_-}{\sqrt{2}})$, where every bead is the same species and uncharged.

The exponentiated H functional in Eq. 2 possesses a rapidly oscillating phase that give rise to a critical efficiency decrease for traditional sampling methods known as the sign problem [2, 3]. By promoting the fields to be complex variables and employing complex Langevin (CL) sampling [4], the efficiency loss is avoided. The CL equations of motion used here are:

$$\frac{\partial w_+(\vec{r},t)}{\partial t} = -\lambda_{w_+}\frac{\delta H[w_+,w_-,\varphi]}{\delta w_+(\vec{r},t)} + \eta_{w_+}(\vec{r},t) \tag{6}$$

$$\frac{\partial w_-(\vec{r},t)}{\partial t} = -\lambda_{w_-}\frac{\delta H[w_+,w_-,\varphi]}{\delta w_-(\vec{r},t)} + \eta_{w_-}(\vec{r},t) \tag{7}$$

$$\frac{\partial \varphi(\vec{r},t)}{\partial t} = -\lambda_{\varphi}\frac{\delta H[w_+,w_-,\varphi]}{\delta \varphi(\vec{r},t)} + \eta_{\varphi}(\vec{r},t) \tag{8}$$

where the $\eta_i(\vec{r},t)$ are real-valued Gaussian-distributed white noise random variables with statistics $\langle \eta_i(\vec{r},t)\rangle = 0$, $\langle \eta_i(\vec{r},t)\eta_i(\vec{r}',t')\rangle = 2\lambda_i\delta(\vec{r}-\vec{r}')\delta(t-t')$. Numerically propagating the CL equations of motion generates importance sampled sequences of field configurations, and ensemble averages over field-theoretic operators can be replaced by time averages over field samples by the ergodic principle. Complex Langevin (CL) simulations are performed in reduced units by scaling all length scales by a reference distance $d = \frac{b}{\sqrt{6}}$ which is the pre-factor in the chain-length scaling relation of an ideal homopolymer radius of gyration $R_g = dN^{1/2}$ [5]. In all cases we set the Gaussian smearing length a equal to this reference length scale $a = d$. All CL simulations were performed in a cubic box of length $L = 32d$ using periodic boundary conditions. Fields were sampled with a spatial collocation mesh of 32 [6]. An exponential time difference (ETD) algorithm [7, 8] was used in the numerical propagation of the CL equations of motion with a time step of $\Delta t = 0.05$.

The state of the system is determined by specifying a dimensionless excluded volume parameter $B = v/d^3$, a dimensionless electric field $E = 4\pi l_B/d$, and a dimensionless polymer chain density $C = \rho d^3$ with $\rho = \sum_i n_i N_i/V$ where i indexes indicate the chain type.

Supplementary References

[1] S. F. Doi, M.; Edwards, *The Theory of Polymer Dynamics* (Oxford University Press: Oxford, U.K., 1988).

[2] K. T. Delaney and G. H. Fredrickson, The Journal of Chemical Physics **146**, 224902 (2017).

[3] G. Fredrickson, The Equilibrium Theory of Inhomogeneous Polymers (Oxford University Press: Oxford, U.K., 2006).

[4] J. R. Klauder, Journal of Physics A: Mathematical and General **16**, L317 (1983).

[5] P. J. Flory and M. Volkenstein, Biopolymers **8**, 699 (1969).

[6] P. Anderson and N. Kedersha, The Journal of Cell Biology **172**, 803 (2006).

[7] M. C. Villet and G. H. Fredrickson, The Journal of Chemical Physics **141**, 224115 (2014).

[8] D. Düchs, K. T. Delaney, and G. H. Fredrickson, The Journal of Chemical Physics **141**, 174103 (2014).
